# Controlling emotions—nurses’ lived experiences caring for patients in forensic psychiatry

**DOI:** 10.1080/17482631.2019.1682911

**Published:** 2019-10-24

**Authors:** Lars Hammarström, Marie Häggström, Siri Andreassen Devik, Ove Hellzen

**Affiliations:** a Department of Nursing, Mid-Sweden University, Sundsvall, Sweden; b Department of Health Sciences, Nord University, Namsos, Norway

**Keywords:** Encounters, forensic nursing, forensic psychiatry, lived experience, nurse-patient relationship, nursing, phenomenological-hermeneutic approach

## Abstract

**Purpose**: Nurses working in forensic psychiatry often encounter offenders who have a severe mental illness, which may cause ethical challenges and influence nurses’ daily work. This study was conducted to illuminate the meaning of nurses’ lived experiences of encounters with patients with mental illnesses in forensic inpatient care.

**Methods**: This qualitative study employed narrative interviews with 13 nurses. Interviews were audiotaped and transcribed verbatim and analysed following a phenomenological-hermeneutic approach.

**Results**: Four key themes were revealed: “Being frustrated” (subthemes included “Fighting resignation” and “Being disappointed”), “Protecting oneself” (subthemes included “To shy away,” “Being on your guard,” and “Being disclosed”), “Being open-minded” (subthemes included “Being confirmed,” “Developing trust,” and “Developing compassion”), and “Striving for control” (subthemes included “Sensing mutual vulnerability” and “Regulating oneself”). Further, working in forensic psychiatry challenged nurses’ identity as healthcare professionals because of being in a stressful context.

**Conclusions**: Dealing with aggressive patients with severe mental illnesses threatens nurses’ professional identity. Nurses must attempt to empathize with patients’ experiences and respond accordingly. Utilizing strategies rooted in compassion such as self-reflection, emotional regulation, and distancing themselves when necessary may enable nurses to more effectively respond to patients’ needs.

## Introduction

Forensic psychiatry provides services for offenders with severe mental illnesses (Nedopil, Taylor, & Gunn, 2015). Patients have often committed acts of violence (Rydenlund, Lindstrom, & Rehnsfeldt, 2019). According to Vorstenbosch, Bouman, Braun, and Bulten (2014) one third of the offenders who commit these acts have a severe mental illness, if a diagnosis is established after a forensic psychiatric examination, the person will likely be admitted to forensic psychiatric care, which is characterized by long hospital stays. In an environment that is characterized by security (Doyle, Quayle, & Newman, 2017) and can be experienced as restricted (Olausson, Danielson, Berglund Johansson, & Wijk, 2019). The caring relationship is central to forensic nursing (Encinares, McMaster, & McNamee, 2005) and a major part of care consists of nurses’ encounters with the patient (Tenkanen et al., 2016), and Rask and Brunt (2006) notes that nurses should promote conversations with patients. Patients may receive as little as half hour and 1.5 hours per day for treatment and structured activities, respectively (Sturidsson, Turtell, Tengström, Lekander, & Levander, 2007). Social interactions constitute the remainder of the work according to Rask and Hallberg (2000).

To interact with people in psychiatric nursing creates possibilities to affect patients’ mental illness, and it is a key component of the rehabilitation process (Hellzen & Asplund, 2006). A caring conversation between the nurse and the patient could indicate an improvement in the patient’s health (Rydenlund et al., 2019); however, there is currently scant research concerning these encounters. There is some evidence that healthcare professionals can be judicial; do not listen; and lack the adequate competence to address hopelessness, apathy, anger, and sorrow (Harris, Happell, & Manias, 2015). The fact that the patient has committed a crime may cause stress and frustration, thus damaging the potential relationship between nurse and patient and fostering mistrust (Harris et al., 2015).

Forensic psychiatric care is complex, regardless of whether the care is viewed as care or control (Kettles & Woods, 2006; Maroney, 2005). Little is known about how nurses respond to patients’ experiences (Myklebust & Bjorkly, 2019). Løgstrup (1997) presented a phenomenological-hermeneutic ethical demand—he stressed that encounters with other people come with a distinct responsibility: people exist together and are dependent on each other. People extradite themselves to a “me and you” relationship. Examples of such actions are to show trust, mercy, openness, and honesty. When they are absent, it is an indication of a selfish, modern world, and the ethical requirements transform into a duty.

Since the care is characterized by constraint and coercion, patients’ dignity may be offended through objectification (Jacobson, 2009). Rask (2002) stressed that a trusting relationship between the patient and nurse can improve forensic care; however, this requires a deeper understanding of nursing in forensic care. It is known that a caring relationship is of importance, but to what extent and how these encounters unfold in clinical practice is relatively unknown (Goulter, Kavanagh, & Gardner, 2015). There is a plea of nurses to be caring, protecting and trustful (Tingleff, Hounsgaard, Bradley, Wilson, & Gildberg, 2019). According to Hörberg, Sjogren, and Dahlberg (2012), patients in forensic care express that this trusting relationship is missing. The question is, how can forensic care that is custodial and corrective be based on nursing, and how can caretakers equip themselves with the necessary tools derived from nursing and ethics according to Hörberg (2015)?

Meeting other people comprises a permanent fusing between understanding and impression by establishing trust in inter-human relationships. Meeting another person comes with expectations—an anticipation that the other will receive us and fulfil our expectations. Løgstrup (1997) posits that, if the expectation is not received, there is a risk of meaninglessness. It is necessary to evaluate the care from the nurses’ perspective (Selvin, Almqvist, Kjellin, Lundqvist, & Schroder, 2019). Nurses must be supported so that they provide care that eases patients’ suffering and prevent future crimes. If feelings like fear, disorientation, and anger become the foundation of care, the caretakers will not be able to ease patients’ suffering (Sjögren, 2004). Nurses endeavour to make patients submiss to the care, thus becoming manageable and displaying positive behavioural adaptation. According to Hörberg (2008), the complexity of forensic care is that nurses’ tasks are contradictory—they are supposed to care, guard, and protect; connect with the patient; create a trusting relationship; ease the patients’ suffering; and improve their health and wellbeing. Letting a patient’s expressions become the nurse’s impression, confronting the nurse with the risk of letting intuition and emotions affect his/her caregiving (Devik, Enmarker, & Hellzen, 2013). The aim of this study was to illuminate the meaning of nurses’ lived experiences of encounters with patients with mental illnesses in forensic inpatient care.

## Materials and methods

Qualitative research involves studying things in their natural setting, attempting to make sense of, or interpret, phenomena and the meanings people bring to them (Creswell & Poth, 2018). These meanings constitute individuals’ lived experiences and can be expressed through reflection on actions in narratives (Lindseth & Norberg, 2004) of nurses encounters with patients with mental illnesses in forensic inpatient care.

### Procedure and setting

Narrative interviews were conducted with 13 participants, based on a model of sample size in qualitative selection and information power (Malterud, Siersma, & Guassora, 2015). All participants worked at a forensic hospital in Sweden. The clinic consists of approximately 180 employees and 100 patients. Most patients are men aged 25–45 years who were convicted of some sort of violent crime. Approximately 60% of patients have schizophrenia or another psychotic disorder. An invitation to participate was mailed, with written information about the study and a consent form, to the heads of the clinic and each ward. Study approval was obtained by the head of the clinic.

### Participants and data collection

A purposive sample was recruited among nurses with experience of caring for patients with mental illnesses in forensic inpatient care. The interviews were conducted at the forensic clinic, at a preferred place chosen by the participants. Participants were 10 men and 3 women (median (*Md*) age = 36 years, age range = 28–67 years). Participants had worked in forensic psychiatric care between 5 and 46 years (*Md* = 11 years), and there were 5 registered nurses, among those 3 specialist nurses in psychiatric care and 8 assistant nurses, all with special training in psychiatric care.

In the presentation of the results, all staff are referred to as “nurse” to conceal their identities. Data collection was conducted through recorded, individual, and narrative interviews with open-ended questions (Mishler, 1986). Participants were asked to narrate their lived experiences of encounters with patients with mental illnesses in forensic inpatient care. The interviews lasted from 41 to 60 minutes (*M* = 48 min). The main questions included, “Can you tell me about an encounter with a patient that evoked negative feelings?” and “Can you tell me about an encounter with a patient that evoked positive feelings?” Further questions included, “How did you feel?,” “Can you tell me more?,” and “Has that happened before?.” The first author transcribed the interviews verbatim.

### Phenomenological-hermeneutic approach

The interview text was interpreted using a phenomenological-hermeneutic approach (Lindseth & Norberg, 2004). The process of interpreting the text goes through three phases: naive understanding, structural analysis, and comprehensive understanding. During the first phase, the naive understanding the text was read many times with an open mind; this was to get an overall awareness of the text, which ends in a formulation of the initial understanding of what the text is about. The second phase, the structural analysis, is a more precise form of analysis to recognize parts and patterns and to seek clarification of the text through outdistance and a critical way of being. This was achieved by analysing all the meaning units, which was sorted into themes and subthemes. The last phase of analysis was the comprehensive understanding, which is a form a dialectic movement between explanation and understanding; it is a way of seeing the whole considering its parts, and the parts considering the whole. It is an analytical, in-depth interpretation of all three phases. Altogether, this interpretation produces a comprehension of what the whole text represents. The process of interpretation is not linear; rather it is a spiral, dialectic movement between the parts.

### Ethics

All participants received information about the research both orally and in writing. All participants provided written consent, which was stored by the first author. Participation was voluntary, and all interviewees were guaranteed confidentiality. All participants could, at any time, cease participation. All participants were provided with the first author’s and supervisors’ contact information. Ethical approval was obtained by the regional ethical review board (no. 2018/157-31) and was conducted per the Declaration of Helsinki (WMA, 2008).

## Results

### Naive understanding

During their work, nurses face various patient expressions. The encounters are based on nurses’ willingness to do well; however, they are sometimes characterized by violence, resistance, and threats—thus creating various obstacles that arouse feelings of frustration, disappointment, fear, and humiliation. Contrastingly, encounters can also be positive, evoking feelings of competence, compassion, satisfaction, pride, trust, and pleasure concerning patients’ recovery.

The text implies that encounters with patients who commit serious crimes can be arduous to understand and difficult to navigate for nurses, owing to the long-term care and ambivalence that occurs because of the diverse aspects of care including protecting society and doing what is best for the patients. These opposing views are also described as a potential source of conflict—a conflict based either on caring and alleviating suffering or on guarding and fostering patients. However, letting patients’ expressions make an impression and thus sensing their vulnerability can guide the nurses in regulating their own feelings.

### Structured analysis

Multiple structured analysis resulted in four themes and ten subthemes, see Table 1. The presentation of the essential meanings of the phenomenon—nurses’ encounters with mental ill patients in forensic inpatient care—is written in present tense and describes how the phenomenon is; i.e., the meaning and not what the participants said about it.

**Table I.** Overview of themes and subthemes.

Being frustrated	Fighting resignation Being disappointed
Protecting oneself	To shy away Being on your guard Being disclosed
Being open-minded	Being confirmed Developing trust Developing compassion
Striving for control	Sensing mutual vulnerability Regulating oneself

### Theme 1: being frustrated

Being frustrated means being upset about one’s limitations as a nurse concerning what they want and what they can do for the patient. It includes feelings of seeing oneself as strong, taking on responsibility, acting alone, and coming to short for unattainable demands. The feeling of perplexity around what it means to care for forensic psychiatric patients is strong. Nurses sometimes do not know how or what to do or say to reach the patient, except that they know what the patient has done is wrong, illegal, and totally unjustifiable. This theme consists of two subthemes: fighting resignation and being disappointed.

#### Fighting resignation

Not having anything to orient themselves to after the care creates a sense of perplexity, resignation, and hopelessness—despite repeated attempts to reach patients.

“Hopelessness isn’t an unusual feeling; it can come over you sometimes. It can come when you don’t know how to respond to someone in order to get your meaning across. This may feel dull when trying to reach them; it sometimes feels hopeless when you have tried so much, and nothing works.”

Nurses may feel despair when they do not obtain positive results. Nurses also become confused when they assess a patient as ready to leave the clinic but the patient does not manage to integrate back into society. They feel indecisive and seek explanations for why.

“It can be a hassle with a patient group that would not need to be cared for in our clinic. It often is so anyway because of all the relapses, which leads to an extension of care times. It’s not so much an annoyance as a sense of hopelessness. But it’s special here—with the long times of care.”

Long-term care may result in a sense of powerlessness and lack of control among nurses since they do not see the results of their work in patients’ development. Coming up short of unrealistic demands and not reaching expectations feels wrong and reinforces feelings of hopelessness. Being part of an environment that gives what is considered bad care could be demoralizing for the nurse. Not seeing the results of their work despite their ambitions is expressed by nurses feeling anonymous and powerlessness.

#### Being disappointed

Being disappointed means becoming aware of the letdowns that could arise when caring in forensic psychiatric care. It is a challenge to meet the feelings of disappointment with colleagues. The work does not, at times, feel fair. According to the nurses, the fact that they are responsible for care is something that they must “live with.”

“It’s hard to always be the one who says no, and you get upset when the patients are on you all the time.”

Nurses expressed that interacting with individual patients also means having an overall sense of the patient, which he or she brings to the next meeting. Becoming aware that it is not always easy to show understanding towards the patient and that it can be difficult to live up to expectations can evoke anger and a sense of failure.

“I may carry with me some frustration from a previous meeting; then, it might be easier for me to overreact and get frustrated in a similar situation.”

Becoming aware of how to work, think, and act is important for nurses. Nurses expressed that long-term care can affect them negatively, with feelings of frustration due to the patient’s illness that sometimes does not show any improvement. Wanting to help and then being rejected by the patient fosters nurses’ sense of frustration. However, both becoming aware and recognizing their own shortcomings helps nurses cope with this frustration.

“I can link it directly to work effort—how much commitment I have put into this, how many mind maps, how many whiteboard pens I have worn out on this patient, [and] how much feet I have rasped. When I finally present that, ‘I think you and I have come up with the solution,’ the patient responds, ‘that sounds hard.’ Then, the frustration grows.”

### Theme 2: protecting oneself

The theme of protecting oneself includes encounters that evoke negative feelings among nurses. This theme consists of three subthemes: to shy away, being on your guard, and being disclosed.

#### To shy away

Working in forensic psychiatric care means being exposed to patients who have committed heinous crimes, such as violent crimes and sexual abuse against children. Nurses explain this as a reason that it is sometimes difficult to approach the patient and that they are uncertain in the care they must provide.

“I feel like I’m getting angry. He tried to murder a little girl in the most brutal way I heard of. There goes my own limit to be able to feel goodness and love. I could not care for him in the same way I care for other murderers. For me it was the exception.”

Nurses know what the patients need; however, they cannot satisfy it. Instead, the situation is handled by avoiding contact with the patient. The nurses expressed that, whatever they do, everything feels wrong.

“There’s one patient where I find it difficult to feel goodness and love. It’s a patient that I thought was horrible. He was a pedophile. Many considered him the best patient. Because he did exactly what you said. [He] handled himself perfectly; but, I could not see him in the eyes. I just said hello and what was necessary.”

Distancing also resulted from nurses feeling that patients were a threat and that they could lash out in violence. This fear led to uncertainty in their care, which affects nurses’ actions and the caring relationship. This also led to contradictory emotions, because sometimes nurses are not prepared to pay the price that it can cost to handle their feelings.

“It’s worse when you get scared. When you feel that you’re about to get hit, or when you feel that the whole situation is threatening, you have to put down all of your energy to be able to cope with the situation.”

#### Being on your guard

Working as a nurse in forensic psychiatry means being on your guard. There is a nagging feeling that, at any time, a situation can occur. To prevent dangerous situations the nurse needs to be constantly on guard. Sometimes the patient’s history and professional experience help the nurse stay one step ahead.

“With some patients, you have to be on your guard. You don’t let them behind your back. Such times, it’s not entirely safe to be at work. You know what they have done. Perhaps they have a history of being rowdy.”

“For some reason, I got a weird feeling when he walked by. Something wasn’t right—a gut feeling. So, I turned around because I felt he was behind me. He was one of those who you needed to have your eyes on.”

New patients create uncertainty because there is a lack of knowledge about the patient. Therefor they need to proceed with caution when encountering new patients. Sometimes the distrust is linked to the patients’ experiences. The nurses work experience indicates that a frightened patient may be dangerous.

“[It] reminds you of that one time a patient attacked you, for example. It also means that you might be a little more cautious when you are going to meet this patient. Maybe you keep a little more distance because you do not feel completely safe with him. I didn’t know him. [I] didn’t know what he was capable of. He was very wound up and frightened.”

#### Being disclosed

Being humiliated by a patient can affect nurses, especially if it occurs frequently or in front of other people. Feeling disclosed is hard to defend against and may make nurses lose their composure.

“I think this person has found a sensitive point in me—where I am somehow vulnerable or become offended. Sometimes I can withstand almost anything while sometimes I can withstand almost nothing; those times I will be sincerely offended.”

Being unable to change the situation was problematic for nurses. Nurses want to handle the situation and stand up for themselves. Instead, there’s a feeling of being disclosed and an inability to act like they do with other patients.

“I took it personally. A thing that normally does not concern me; but, he managed somehow to get under my skin.”

### Theme 3: being open-minded

Being open-minded refers to nurses’ ability to understand patients’ history and disease. It is needed for an effective patient–nurse relationships. This theme consists of three subthemes: being confirmed, developing trust, and developing compassion.

#### Being confirmed

Being confirmed means that nurses’ engagement increases. The feeling of not being alone in the situation decreases when nurses receive validation from their colleagues. They feel that they are easily understood by colleagues who have similar experiences in forensic psychiatric care and thereby understand the caring relationship. This evokes a willingness to provide care.

“I think it matters for my commitment. I think we, in the staff group, are still quite good at … when someone manages to do something good. That you get to hear it. I can feel, that if I receive praise, [that] it’s something I want to maintain.”

Patients’ expressions of gratitude contribute to the caring relationship. The nurses appreciate when the patients value good nursing care, which increases nurses’ self-esteem and fosters continued commitment. Long-term forensic care with severely ill patients is so arduous, that when there is success with a patient, it brings nurses joy and validates the work they do.

“The patients don’t show much gratitude; but some do it. Then, you get energy and a great feeling in the body. Then, I feel that you’re doing something meaningful.”

#### Developing trust

Developing trust is vital for the caring relationship and means having the courage to open up to the patient and taking the patient seriously. Showing confidence in the patient means that the relationship becomes predictable.

“If I can trust the patient I also dare more—like, sharing myself. When I learn how to respond to him, I feel more secure. Then you dare more. I can’t feel the mood of the patient if I go around being scared or don’t feel safe.”

Trust also means that the balance of power is reduced by the patient’s participation. Knowing the patient may also mean being together, through that the distance and paternalistic relation between the two parties is decreased.

“For me, I think the key has been that I managed to create trust through my encounters. You should meet his needs and listen. He should feel involved.”

#### Developing compassion

Letting the patient make an impression means seeing “the person” and not just “the patient.” Becoming aware of and recognizing the vulnerability of the patient’s situation characterizes these interactions.

“She then told me about her whole life. To hear about how her life has been—about why she committed the crime she had done. It all felt very tragic.”

It is not only the patient’s life that makes an impression on the nurse; the patients’ temperament is also of importance. If the patient is perceived as a child, the nurse may find it easy to provide care since feeling sorry for the patient evokes empathy.

“All of a sudden, he begins to cry. It all became very different very suddenly. You felt how frightened he was—how small he was. I felt very sorry for him.”

Reflecting on the patient’s expressions over time promoted a deeper relationship between nurses and patients. Which may also create a more caring relationship.

“I’ve been with these patients for several years. I’ve established stronger ties with them. Thus, I become more personally involved in them. Over time, it has surely become so that I maybe care more about them.”

### Theme 4: striving for control

This theme consists of two subthemes: sensing mutual vulnerability and regulating oneself.

#### Sensing mutual vulnerability

Sensing mutual vulnerability refers to the feeling that occurs when the nurses are affected by the patients’ expressions. Nurses feel vulnerable when they perceive patients’ vulnerability. It was described as frustrating when patients’ wellbeing and health were at risk. Feelings of sadness and loneliness affected patients, which seemed to arouse a sense of compassion among nurses. Nurses used intuition and empathy to guide their responses to some patients.

“I feel it’s tough; [it is] hard to be among these patients because they feel so amazingly bad. I already felt before that he was afraid. It was as if he felt crowded. He could not flee even if he wanted to. He could just as easily become aggressive to deal with the situation.”

#### Regulating oneself

Regulating oneself refers to nurses’ responsibilities, including legally, concerning patients’ care. Care is described as special, because the patient group has complex problems and partly because the institution environment is characterized by a high level of security. In nursing care, the nurses must balance between patients’ rights and the safety of society. The care is complicated as patients may be ill, aggressive, and provocative.

“If they’re being aggressive, then you’ve to stop and think before going into a situation. Some may be so provocative; but, if you can find your own sense of security and calm—if it’s from colleagues or whatever—the encounter with the patient will be better. You have to keep track of your own feelings in order to take care of someone else’s feelings.”

If nurses are unable to cope with their feelings, there is a risk that they will lose control of themselves and the situation. Regulating oneself in such a situation means, if possible, taking a step back to finding room to take a breath.

“If I am overcome by emotions and find myself losing control, I try to pull myself out of the situation until the level of affect decreases. Then I can ponder the situation [and] think about what has happened—why did I react so strongly? I would say that the feeling that is most difficult for me to distance myself from, or to regulate, is fear. Anger is, in a way, more manageable.”

### Comprehensive understanding

The overall interpretation is based on the authors preunderstanding and naive understanding, themes, subthemes, and reflecting upon them in relation to the research question, context, and literature. The meaning of encountering patients with mental illness in forensic inpatient care is characterized by the asymmetric relationship between the nurse and the patient. This constitutes a fundamental moral challenge that nurses must cope with. For the nurses, the encounters involved being frustrated, protecting oneself, being open-minded and striving for control. Patients’ expressions of threat, violence, and provocative behaviour threaten nurses’ professional identity. Nonetheless, nurses attempted to empathize with patients’ experiences and displayed competence in assessing patients’ expressions. Nurses placed themselves in a vulnerable position by acknowledging patients’ uniqueness and individual needs. This strategy fostered self-reflection, situational assessment, and compassion for patients. This allows nurses to control themselves, the patient, and the situation.

## Discussion

The aim of this study was to illuminate the meaning of nurses’ lived experiences of encounters with patients with mental illnesses in forensic inpatient care. We found four themes, further broken down into ten subthemes, that shed light on interviewees’ lived experiences: “Being frustrated,” “Protecting oneself,” “Being open-minded,” and “Striving for control.” We found that patients’ expressions emotionally affected nurses’ caring actions and preferences as well as their professional self-esteem and moral identity.

Working in a forensic environment challenges nurses’ identity as a healthcare professional because of their obscurity and vulnerability in stressful work situations. Moral distress is an inherent risk in forensic psychiatric care with its complex patient group that have varied problems. This leads to an institutional environment characterized by a high level of security in the interface between law and psychiatry (Carroll, Lyall, & Forrester, 2004). Nurses may experience distress when dealing with their own fear owing to patients’ potentially provoking and violent behaviour. This means that the provision of competent and compassionate care can be compromised by nurses’ fear and lack of knowledge. The care approach may also fail owing to a lack of self-confidence or courage in nurses’ interactions with patients. However, this is a balancing act, and power is an underlying issue. Nurses are empowered by their expertise and their mission but disempowered because they must try to adjust to patients’ complexities.

Working in forensic psychiatry means providing nursing care for long periods. Patients who are calm are often perceived as accepting of the care given and following the rules according to Eivergard, Enmarker, Livholts, Alex, and Hellzen (2018). Nurses often expressed ***being frustrated*** by not being able to reach patients or make progress in their treatment. A major part of forensic nursing is being firm, setting limits, and defining boundaries, which affect the nurse–patient relationship (Bowen & Mason, 2011). There is always a risk that the encounters are viewed as paternalistic (Hörberg et al., 2012; Norvoll & Pedersen, 2016; Selvin, Almqvist, Kjellin, & Schröder, 2016). Forensic inpatient care is strenuous owing to long hospital stays (Rao et al., 2009), and nurses with negative perceptions may be more likely to provide poor care quality (Kukulu & Ergun, 2007).

According to Jacob, Gagnon, and Holmes (2009), feelings of frustration, disappointment, and resignation can be obstacles that nurses must overcome. These feelings can make nurses doubt their own actions, which becomes clearer if these feeling occur for a long time (Dennis & Leach, 2007). This could also influence patients to experience insecurity and powerlessness. This is because nurses do not see the patient as dynamic individuals; rather, they are viewed as or she is but static, one-dimensional people (Lilja & Hellzén, 2007). According to Olausson et al. (2019), a relationship can be a lifeline—saving the patient from loneliness and contributing to their well-being. If patients sense that nurses are attempting to empathize with them, it may foster trust and open the lines of communication. Strengthened by the ethos of caring and ethics, nurses must engage with patients, which too will promote trust (Rydenlund et al., 2019).

The theme ***protecting oneself*** refers to nurses’ lived experiences concerning facing the unpleasant. Nurses expressed a distance between themselves and the patients as a way of dealing with mixed emotions that arises when caring for patients who committed despicable crimes. However, nurses must strive to look beyond patients’ crimes and backgrounds, instead focusing on support and recovery (Bowring-Lossock, 2006). This is difficult when feeling unsafe or afraid, which negative affects the caring environment (Leutwyler & Wallhagen, 2010). Being exposed to threats, violence, and provocative behaviour was described by all the nurses, and this can contribute to a distance in the nurse–patient relationship. Consistently, patients’ ability to follow the rules and not showing aggressive behaviour is deemed acceptable patient behaviour in the eyes of nurses (Eivergard et al., 2018).

Results from this research indicate that within a highly regimented context, nurses are socialized to incorporate representations of the patients as being potentially dangerous. Thus, they distance themselves from idealistic conceptions of care. The results also emphasize the implication of fear in nurse–patient interactions, particularly how fear reinforces nurses’ need to create a safe environment. This results in a consistent negotiation between risk and security, in which nurses are forced to scrutinize their actions and preventing nurses’ future engagement. This can be contradictory since not knowing or uncertainty about a patient is a source of feeling unsafe. Continuity and being present leads to the patient feeling safe in the nurse–patient relationship, which creates opportunities to further establish a good relationship and, in turn, increases the ability to make the patients’ needs visible (McCann & Phillips, 2007).

Our findings showed that not knowing the patient makes it unpredictable and difficult to determine how to best care for the patient, which was also suggested by Holmes, Murray, and Knack (2015). Forensic psychiatric care is a restricted environment, and beyond what is considered a “normal” life (Olausson et al., 2019). Patients express a longing for encounters in which the nurse is the one taking a step forward and not disappearing when times are tough (Lindström, 1995). Not feeling safe also fosters hopelessness, and it can be an obstacle to understanding patients’ views on health, wellbeing, and existence. A state of being non-judgemental, present, and open towards the patient is desirable for forensic nurses (Bowen & Mason, 2011).

Nurses’ can facilitate patients’ path to health by expressing a willingness to understand patients’ experiences (Rydenlund et al., 2019). In forensic psychiatry, fear is considered a part of the milieu. Nurses present a front—not showing fear as a way of dealing with these emotions. It is a self-protective strategy that is necessary to see the patient as a human being, regardless of whether the patient is evoking fear. This is considered an obtainable professional value (Jacob & Holmes, 2011).

Like all nursing care, nursing practice in forensic psychiatry care is grounded in ethics, and the ethical responsibilities underlie all nursing interactions towards individuals, next of kin, and colleagues (ICN, 2014). When caring for a patient who emotionally touches them, it was revealed that the nurses were opened themselves to patients’ vulnerability. Expressing sympathy towards patients and ***being open-minded*** are critical aspects of nursing care.

Developing compassion can promote nurses to be fair, respectful, consistent, and knowledgeable in their encounters with patients (Maguire, Daffern, & Martin, 2014). The nurse–patient relationship in forensic psychiatric settings should be grounded in trust and confidence, and patients require opportunities for emotional reconciliation, as suggested by Salzmann-Erikson, Rydlo, and Wiklund Gustin (2016). Being a nurse in forensic psychiatry is a complex role, and there is a tension between maintaining safety and promoting a therapeutic and patient-centred approach (Green, Shelly, Gibb, & Walker, 2018). Nurses strive to maintain professional boundaries and aspects of therapeutic communication, including establishing “trust” and “validation,” according to Doyle et al. (2017). Establishing a trusting relationship with patients in forensic psychiatric settings is viewed as a less oppressive way to control patients and guide them in directions that are preferable for the nurses and for society. This could be achieved through encounters in which the gap between the patient and the nurse is reduced (Salzmann-Erikson et al., 2016). Encounters in forensic psychiatry invite nurses to carry the burden of guilt and suffering during long periods (Rydenlund et al., 2019), which our findings suggest is made possible by having trust and compassion.

Our findings also suggest that nurses were often emotionally affected by patients’ expressions of threat, violence, and provocative behaviour. This situation forced a nurse, when the situation become too severe, to take a step back and distance him/herself from the patient as a way of ***regulating oneself.*** Sometimes forensic nurses need to distance themselves from their patients because of policies or procedures around control (Gillespie & Flowers, 2009). It is a challenge for nurses to maintain a positive relationship with patients, especially when they have been threatened, harassed, insulted, or physically injured by a patient. Nurses must move past their own feelings towards the patient and attempt to help the patient regain trust in them to preserve their relationship (Holmes et al., 2015).

Nurses described that encounters also means facing suffering and their own reactions to it. Trying to maintain a positive relationship through encounters that vary from “normal” everyday circumstances, nurses must look past the problematic behaviours and understand that what they are seeing in patients is the illness and not actual “bad” behaviour, as described by Holmes et al. (2015). Our findings show that, when nurses understand this, they can see beyond the façade. This enables nurses to detect patients’ conceptions of themselves. This indicates that the caring encounter is formed by patients’ needs. The nurses described that facing patients’ suffering sometimes required that the nurse take a step back—not to abandon, but rather to come closer, which is also described by Vincze, Fredriksson, and Wiklund Gustin (2015).

The findings in our study can be understood considering the Danish philosopher Løgstrup (1983), who stated that vulnerability is a fundamental condition of human life. Our findings indicate the importance of nurses’ vulnerability when participating in another’s life. According to Løgstrup (1997), meeting another’s expressions is also ontologically, an inter-human act where each one of the actors turns to each other in a person-to-person encounter in which they are guided by their perception and vulnerability. For nurses, the challenge is also how professionalism plays in to their interpretation of the situation. Our findings indicate that in some occasions the caring encounter is formed by the patients needs and the nurses ability to regulate their own expressions. Regulation means inviting the patient into the “room of awareness,” where the two parties meet each other and themselves (Devik et al., 2013). Through regulation, nurses can open the “door” to the “room of awareness” and let the patient’s expression make an impression without hiding behind the protection of one’s own foreknowledge and a stereotyped view of forensic psychiatry patients.

Logically, the “key” to the “room of awareness” is nurses’ ability to interpret patients’ expressions. According to Løgstrup (1983), interpretation is in the movement between perception and understanding.Our findings clearly indicate that nurses’ perception were affected, which was also seen in other studies (Vincze et al., 2015). According to Løgstrup (1978), understanding can also create a distance and is linked to our preconceptions, where cultures and knowledge are embedded. The nurse is moved and affected, present in the perception of something that does not leave him or her untouched (Vincze et al., 2015).

In other words, being present with the patient is challenging when the nurse is confronted, not only with the patient’s expressions, but also with their own reactions. If a nurse encounters a patient with his/her prior knowledge and with a preparedness to categorize what has been said, the “room of awareness” may become locked (Hellzen & Asplund, 2002). Encountering patients’ expressions may be a painful and frightening experience; however, nurses must have the courage to stay with the patient and evaluate both their own and patients’ safety. One way to deal with situations like this is to narrate their experience of patients’ behaviour without questioning it. Instead a true interest, manifested as an effort to understand the patient’s experience and encourage openness of feelings, is awoken in the nurse. As Løgstrup (1983) states, interpretation of sensation is a way of being in the world, sensing openness to people who wanting us something. This means that in the interpretation the nurse experience what it means to be not only human in the world but also how she relates to what touches her in the perception, an appeal of caring for the other based on compassion.

In conclusion, to care for patients within forensic psychiatry means facing numerous of situations that threatens the nurse’s professional identity. Letting the patient’s expression make an impression, taking a step backward to be able to take a step forward by regulating own emotions. Such strategies, creating a temporary distance, enables nurses to come closer to the patients to be able to alleviate suffering, despite sometimes facing threats, violence and humiliation, making decisions based on compassion and the patient’s needs.

### Methodological considerations

Trustworthiness depends on truthful narratives of lived experiences (Lindseth & Norberg, 2004). The first author was known to most of the participants, which hopefully meant that the participants could speak truthfully and freely. On the one hand, this might facilitate trust and that participants could speak freely and truthfully. On the other hand, this could cause participants to be cautious and afraid to reveal weaknesses. On the third hand, it also challenged the first author’s pre-understanding and ability to discover implicit messages. To overcome this obstacle, the author tried to be attentive and ask questions so that new and unexpected elements could be revealed. The first author has strived towards self-awareness of which has been encouraged through self-reflection and discussions with the other authors. The first author conducted all interviews, transcribed the text, and conducted initial analyses. Some of the other authors lacked firsthand knowledge in forensic care and contributed with contesting throughout the analysis. However, all authors contributed significantly to this manuscript. The two main questions were formalized as either positive or negative, which could suggest that the narratives from the participants could be affected in either direction, which could also mean that some encounters or a certain group of patients were forgotten. By requesting both negative and positive experiences from encounters, the intention was also to trigger the participants’ memory when narrating. It may be easier to remember experiences that have been emotional touching, and asking for specific incidents is a recognized technique when working with narrative inquiry (Drew, 1993).

This article does not present an absolute truth or distinct evidence; rather, it can shed light on nurses’ lived experiences of encounters with patients with mental illnesses in forensic inpatient care. Hopefully, it will encourage more research concerning how patients’ expressions impact nurses and the care they provide. It should be considered that forensic psychiatry is governed and controlled by laws that may differ nationwide; therefore, the current results should be viewed as lived experiences in the Swedish context.
